# CAEv–A program for computer aided evaluation

**DOI:** 10.1155/S1463924691000317

**Published:** 1991

**Authors:** W. Bablok, R. Barembruch, W. Stockmann, P. Brauer, P. Graber, R. Michel, D. Vondersehmitt

**Affiliations:** 1 Boehringer Mannheim GmbH Sandhofer Strasse 116 Mannheim 31 D6800 Germany; 2 Zentrallabor. der Med. Univ. Klinik Würzburg D8700 Germany; 3 MB Data Control A G Stansstad CH6362 Switzerland; 4 Universitätsspital Zürich CH8091 Switzerland

## Abstract

The evaluation of new reagents and instruments in clinical chemistry leads to complex studies with large volumes of data, which are difficult to handle. This paper presents the design and development of a program that supports an evaluator in the definition of a study, the generation of data structures, communication with the instrument (analyser), online and offline data capture and in the processing of the results. The program is called CAEv, and it runs on a standard PC under MS-DOS. Version 1 of the program was tested in a multicentre instrument evaluation. The concept and the necessary hardware and software are discussed. In addition, requirements for instrument/host communication are given. The application of the laboratory part of CAEv is described from the user's point of view. The design of the program allows users a high degree of flexibility in defining their own standards with regard to study protocol, and/or experiments, without loss of performance. CAEv's main advantages are a pre-programmed study protocol, easy handling of large volumes of data, an immediate validation of the experimental results and the statistical evaluation of the data.


Journal of Automatic Chemistry, Vol. 13, No. 5 (September–October 1991), pp. 167–179

### CAEv—A program for computer aided evaluation

**W. Bablok, R. Barembruch, W. Stockmann**
*Boehringer Mannheim GmbH, Sandhofer Strasse 116, D6800 Mannheim 31, Germany*

**P. Brauer**
*Zentrallabor. der Med. Univ. Klinik, D8700 Würzburg, Germany*

**P. Graber, R. Michel**
*MB Data Control AG, CH6362 Stansstad, Switzerland*
**and D. Vonderschmitt**
*Universitätsspital, CH8091 Zürich, Switzerland*

*The evaluation of new reagents and instruments in clinical chemistry leads to complex studies with large volumes of data, which are difficult to handle. This paper presents the design and development of a program that supports an evaluator in the definition of a study, the generation of data structures, communication with the instrument (analyser), online and offline data capture and in the processing of the results. The program is called CAEv, and it runs on a standard PC under MS-DOS. Version 1 of the program was tested in a multicentre instrument evaluation. The concept and the necessary hardware and software are discussed. In addition, requirements for instrument/host communication are given. The application of the laboratory part of CAEv is described from the user's point of view. The design of the program allows users a high degree of flexibility in defining their own standards with regard to study protocol, and/or experiments, without loss of performance. CAEv's main advantages are a pre-programmed study protocol, easy handling of large volumes of data, an immediate validation of the experimental results and the statistical evaluation of the data.*

### Introduction

The evaluation of new reagents and instruments in clinical chemistry requires increasingly complex studies with short completion times. The experiments, when carried out on modern instruments, produce data that are very time consuming to process because of their volume and their complicated structures.

National and international committees for standardization in clinical chemistry—like the Commission 'Validation of Techniques' of SFBC (Société Francaise de Biologie Clinique) [1] or ECCLS (European Committee for Clinical Laboratory Standards) [2]—have produced proposals for evaluation protocols and data acquisition, as well as publishing guidelines for the assessment of diagnostic systems [3]. Other authors [4, 5] have presented recommendations for the selection of laboratory instruments. Concepts for developing ways of automatic data acquisition, or programs for the evaluation of new reagents and instruments have also been described. However, a general approach to solving this problem does not seem to have been published. It is obvious that any development of an integrated evaluation system has to rely on the resources offered by computers and data processing techniques, especially since every modern instrument is equipped with its own microprocessor.

Those working in clinical chemistry, who are responsible for designing and carrying out studies on the technical and diagnostic properties of new products, will appreciate software that simplifies the definition of experiments, communication with the instrument, data transmission and result processing. The authors have considerable experience in the evaluation of diagnostic systems [6–8] and have devised a concept for the development of a ***C**omputer **A**ided **Ev**aluation* program CAEv. The following are the targets that were set for the program:

(1) Definition of experiments based on a study protocol, and generation of the corresponding data structures.
(2) Instrument/host communication: online transfer of requests for a particular experiment to the instrument and of results from the instrument.
(3) Immediate validation and evaluation of the experimental results.
(4) Offline editing facilities to input or correct data.
(5) Complete study documentation.
(6) User friendliness and utilization of standard hardware and software.

Software that is commonly used in clinical chemistry laboratories covers at best some parts of the required properties, but is not conceptually designed for an integrated solution.

The first version of the CAEv program was tested, after an internal pilot phase, in an international multicentre evaluation study of the Boehringer Mannheim/Hitachi 747 analysis system (abbreviated to BM/Hitachi 747 in the following text) [9].

This paper describes:

(*a*) The design of the CAEv program package
(*b*) The program development including the communication aspects and instrument drivers.
(*c*) The field test for the user's point of view.
(*d*) A general assessment of CAEv in clinical chemistry.

### Program design

In the early design stage of the CAEv system it became clear that the program had to be structured so that it could be operated by several users. It needed to

0142–0453/91 $3 00 © 1991 Taylor & Francis Ltd

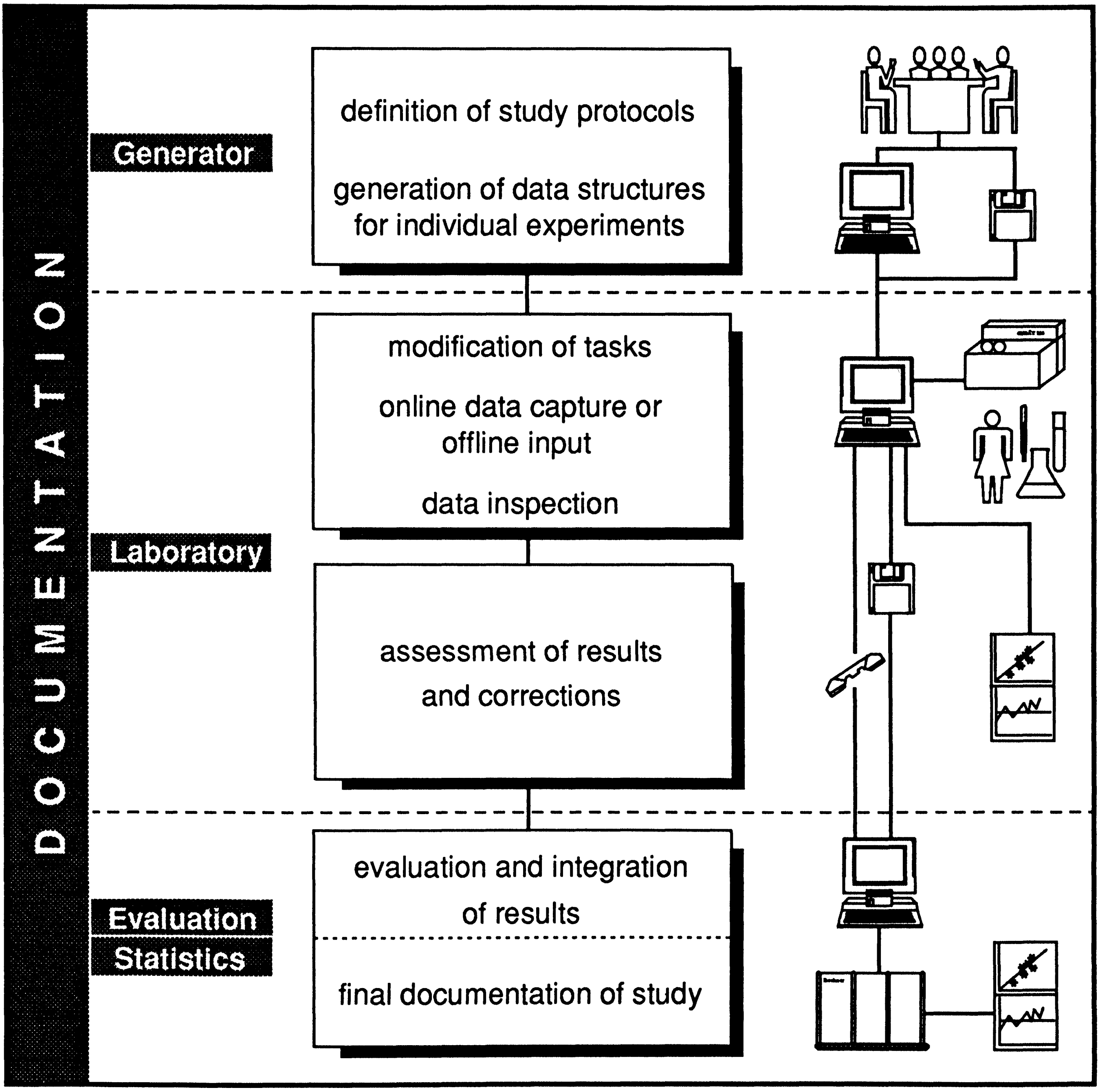

*Figure 1. Graphical presentation of structure and functions of CAEv.*

correspond to the commonly used manual approach in formulating and carrying out an evaluation study:

> Firstly a study protocol is defined and the appropriate data sheets for the documentation and processing of the results are prepared. Then the experiments are performed in the laboratory and the results transferred to the data sheets. Finally, the results are processed manually or by computer programs in order to obtain condensed statistics or graphical presentation of the information to be included in the overall documentation of the study.

Moreover, a study may cover any degree of complexity ranging from one experiment on one instrument in one laboratory to a multicentre study with many experiments using various instruments. As a consequence the program contains subsystems covering generation, communication with the instrument and other computers, and the output of statistics, graphics and documentation (see figure 1). In addition, it offers tools for documentation, which arise in any of the three parts.

### *Generator*

The generator has to provide the means needed for the conceptual phase of a study. There are two approaches to designing such a generator. The first offers a library of evaluation experiments, covering the protocols proposed

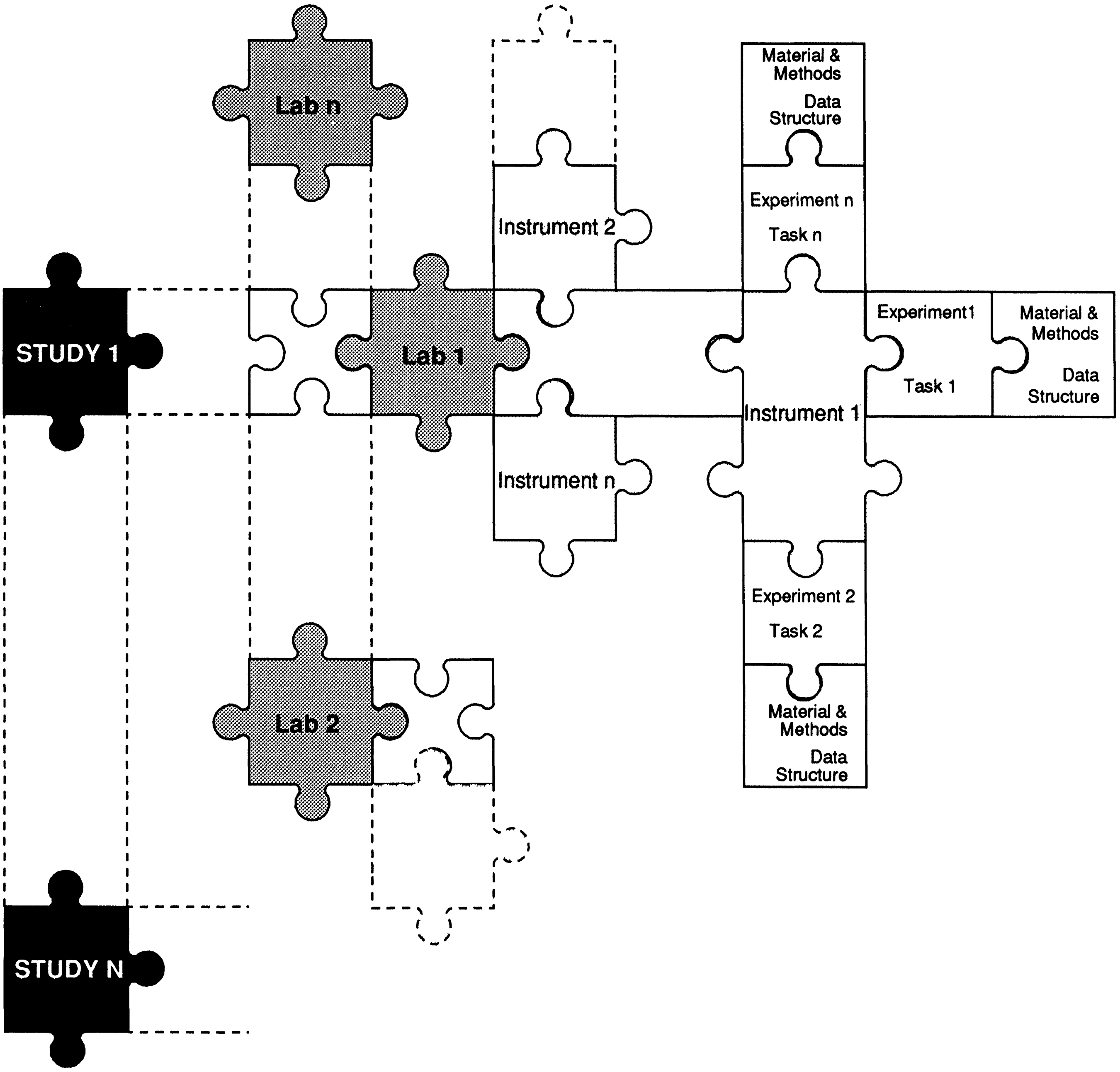

*Figure 2. Components that make up a study.*

by standardization committees (for example ECCLS, SFBC, NCCLS [National Committee for Clinical Laboratory Standards]), and the second allows the user a free definition of an experimental data structure. The first concept is more or less straightforward, asking for the inclusion of additional protocols in the program, if the need arises. The second is more expensive to implement, but permits a high degree of flexibility. The latter was followed and (analogous to concepts and techniques used in software engineering) the generator was designed as a kind of an *evaluator's workbench,* offering a toolkit for the generation of any sort of evaluation study. Its collection of tools and functions should allow any individual definition of a study and of the experiments belonging to it.

At this point it is important to consider the tools needed to define an evaluation study. Figure 2 shows one of several possible arrangements of components that could make up a study; there is no real hierarchy between these components. It is also clear that they do not constitute the final description of a study. Some functionality is needed to fit the pieces together and to produce the data structures which can hold the results from the measuring instruments.

Therefore CAEv offers a set of basic components and a number of functions to be used for the generation of a study. A basic component comprises one or several elements, which the user of the program can define. For example, an obvious member of the basic components is the *'laboratory',* its elements could be defined for instance by *'Lab A'... 'Lab X'* or *'Evaluation Site 1... Evaluation Site 10'.* An example for a function is the selection of elements from different components to define a data structure. The terminology used in CAEv is explained in the next section.

*Basic components*

The following basic components were included as tools for the generator:

(1) Study.
(2) Laboratory.
(3) Test code.
(4) Material.
(5) Task.
(6) Instrument.
(7) Temperature.
(8) Selection.

If necessary, the number of components can be reduced or extended. A basic component can contain a list of one or several elements; for instance, the list of component *instrument* could include two instruments; '*Analyser A*' and '*Analyser B*'. Each element in the list comprises three or more text fields for identification and documentation. The general format to describe an element is given by:

identifier; short text; long text; 1; text 2; . . .

For example, an element in the list of component *instrument* could be represented by the following line:

ID; INST A; Analyser Axxx; . . .

Short and long texts carry the information that appears on the screen or in the documentation; additional text fields can contain information like conversion factors, assigned values, etc. The identifier is used internally to construct codes for data identification.

The program handles all of the editing functions needed for the definition of an element; in particular, it manages the identifier field automatically to ensure a unique code assignment.

The number of entries in a list is not restricted, but there are natural upper limits reflecting the technical possibilities. The components with their list of elements can be used like a library; they are presented by the generator as a menu for editing and/or selection a new study is defined. They are stored in the data base of a study or in a general library. The presentation, editing and selection of elements is one of the various functions in the toolbox.

A short description of the components will explain their usage.

*Study:* CAEv can comprise any number of studies. With the declaration of a new study name a new entry is entered in the component list and will be available for selection until the study is deleted.

*Laboratory:* The laboratory list gives the evaluation sites scheduled for a study. The user of the generator specifies the details. In general, one would have several entries for a multicentre study.

Example of an element:

ID; Lab 1; Laboratory XYZ; A-City; . . .

*Test code:* In CAEv the term 'test code' is used for a conceptually compound object. It can also refer to analytes, reagents and methods of determination, so that the meaning of an element may depend on the context of its usage. For a given study, the list of elements represents all analytes (reagents, methods) which are to be determined in any sample. The short name (a maximum of five characters) is used in the driver (software, which performs the communication between instrument and host computer) for mapping the descriptor of a test code defined in CAEv to the descriptor defined in a particular instrument.

Example of an element:

ID; FRUC; Fructosamine [μmol/1]; . . .

*Material:* The list of elements comprises all sample materials that will be assayed in the study. The description of the material is given in the short and long text. Since instruments may treat different specimens (like serum, urine, liquor) in a different manner, the type has to be specified in the definition of an element. The driver informs the instrument of the actual specimen type, so that the correct settings or predilutions are selected when the sample is processed.

Example of an element:

ID; SPL; Serum-Pool Low; Serum; . . .

*Task:* CAEv uses the terms 'task' and 'experiment' nearly synonymously. However, conceptually a task describes the basic structure of an experiment. For instance, in a linearity experiment (determination of analytical range limits) the task would refer to the dilution sequence, whereas the experiment would include the analytes, for which the limits are to be determined.

The list of elements contains the description for all experiments scheduled for a given study; examples are drift, method comparison, carry-over, reference values and so on.

Example of an element:

ID; Carry-over; Reagent carry-over; . . .

*Instrument:* The list declares the instruments used in a study; the identifier indicates the driver that the generator has to link to the laboratory data files. Data structures that refer to instruments for which a driver is not available can only receive results by manual input. Accordingly, CAEv considers a manual method as an instrument called *manual.*

Example of an element:

ID; HIT 747; BM/Hitachi 747 Analysis System; . . .

*Temperature:* The list reflects different national customs concerning the temperature at which an assay should be run. Within CAEv any temperature value can be defined as long as it can be handled by the instrument and the laboratory.

Example of an element:

ID; 37, 37 degrees centigrade; . . .

*Selection:* An additional tool for the definition of a task is the component selection. It is used to define sublists of basic components. The main application can be seen in the compilation of profiles for test codes and materials. An element in the selection list refers to a subset of elements from a basic component that is specified by the ID field.

Example of an element:

ID; PROF1; Enzyme profile; . . .

### *Generator functions*

The main functions apply to the definition of experiment and study and the generation of corresponding data structures. Also available are maintenance functions, which handle the updating of experiments, the editing of basic components and the manipulation of files.

The compilation of the element lists of the basic components takes place when a new study is defined. The user can refer to lists from the library or other studies and can select, modify, add or delete the elements as the situation requires. The editing process is controlled by the program in order to avoid inconsistencies or ambiguities.

*Definition of an experiment:* In general, evaluation studies will follow more or less closely standardized protocols. There are, however, studies which investigate a special problem or use an individual protocol so CAEv provides tools which allow an experiment to be modelled according to its specific requirements. The modelling takes place on the task level. For a given task the user selects an appropriate sample from the material list, assigns the test codes to be assayed from the test code list and defines the number of replicate measurements. A comment field can be filled in, if necessary, for every sample. This field serves primarily for an additional description of the sample, but can also be used to carry information, like a dilution ratio, for the sample preparation or the result processing.

Additional editing functions allow scanning through the defined samples, insertion, addition and deletion of samples. Thus any desired sample sequence can be defined in order to adapt a task like, for instance, reagent carry-over to specific instrument requirements. The data structure of a task can be developed or changed during the modelling stage of a task. Predefined profile lists for test codes and/or sample material can be used to simplify the definition of a task.

CAEv considers the task as the central element of an experiment as it describes exactly the required data structure in a condensed form. To complete the definition of an experiment the structure has to be expanded in order to conclude the appropriate type of repetition and the instrument assignment.

Three different types of repetition can be requested, two of which are standard, whereas the third has to be defined in the component *selection:*

(1) Series, to define how many independent repetitions of a task should be generated (for example between-day imprecision).

(2) Test code, to define a set of analytes for which the same experiment is to be performed (for example drift, linearity).

(3) Selection, to access a use-defined profile or sublist for repetition (for example interference experiments).

At the end of the definition, the laboratory in which the experiment will be carried out is assigned.

*Definition of a study:* CAEv permits any number of experiments and laboratories to be included in a study. It provides the frame in which all pieces of the study are put together. The main purpose of this program function is the compilation of all experiments that are scheduled for a given laboratory. It outputs a collection of files containing all the information and data structures needed to carry out the study experiments in the laboratory. The files can then be transferred, if necessary, to another computer, which handles the laboratory part of CAEv.

The integration of the results from the different laboratories is controlled by the definitions laid down in this part of the program. This applies also to the study documentation, whose elements have to be declared at this stage.

### *Laboratory application*

After completion of the study definition, all the information needed to perform the experiments in the laboratory is available. Until this point the choice of the hardware, on which CAEv should run, is rather arbitrary. However, the need for online communication with an instrument imposes a number of restrictions, if flexibility is to be maintained. It requires hardware that is generally available, is easy to move and offers good communication facilities. An obvious choice in such a situation is the use of standard PC hardware using MS-DOS as the operating system. The serial port of any PC provides either an immediate, or easily adaptable hardware interface to most of the instruments presently in use. In may laboratories, a PC is already available for normal data processing activities; it could be complemented by a portable laptop PC, if necessary.

To use CAEv on a PC in the laboratory suggests implementing the complete software package on this hardware platform. Since the study generation may be run on a different PC from the laboratory part, the program has to provide several entry points and facilities to transport the data.

Considering the functional properties required for a program in the laboratory environment, one would expect to find at least the following features:

(1) User guidance.

(2) Revision of tasks/experiments.

(3) Assignment of experiments to a run on an instrument.

(4) Online transfer of sample requests and data capture.

(5) Inspection of results.

(6) Input and editing of data.

In addition, communication with a specific instrument should be independent of the program code or the data structures that carry the experimental information. This ensures that a new instrument can be accessed by the program as soon as an appropriate communication driver becomes available.

*User guidance*

So that it is easy to use in the laboratory, CAEv provides support at various levels of the program. First, it presents an overview of the experiments that have to be carried out, together with information on the status of their corresponding tasks. The present version has five different states: open, done, cancelled, open (previously cancelled), done (previously cancelled). Figure 3 shows part of a typical status report from a study.

Secondly, it produces, either on screen or through the printer, an output of the request list that the driver transfers to the instrument. The operator can use this information for preparing the samples in the correct sequence, if direct sample identification is not possible (for example reading a barcode).

Finally, CAEv offers context-specific help screens, which give information on the use of the program and on the study. Help screens referring to a specfic study have to be created individually. The user can decide to present information based on standard protocols or information, which gives the details of the current study. The study help screens have to be prepared and compiled in the generator part of the CAEv. Obviously, a study help is of value only in multicentre trials.

*Revision of experiments*

In general, experiments (tasks) should only be changed to a limited extent in the laboratory. This is justified by the philosophy on which CAEv is based. A study design comprising only one laboratory can easily be modified in the generator part if access to this function is available. A multicentre study may allow only minor variations in the experiments in order to yield comparable results for statistical condensation. Consequently, one can only add additional samples and/or test codes to a task or increase the number of series within one experiment. Cancellation of some or all series of an experiment is also permitted.

*Executing a run on an instrument*

Before an experiment can be run on a particular instrument with the support of CAEv, the user has to go through a number of actions on both the PC and the instrument:

(1) Define the contents of a run that may include one or several experiments (for example one series from a between-day imprecision could be followed by a series from a ring trial and method comparison experiment).

(2) Prepare the samples according to the run request list.

(3) Start the instrument and check the driver for a faultless communication with the instrument (series lines and test code mapping).

(4) Start the transfer of the requests from the PC to the instrument.

After the measuring process the results are transferred to the PC and stored in the appropriate data structures. The transfer can be carried out in real time or batch mode, depending on the facilities of the instrument and the choice of the operator. All instrument-specific activities are covered by the individual driver; the standardized interface to the main program is a common feature in every driver.

*Inspection of results*

One important characteristic of CAEv is the ability to inspect the data, immediately after the completion of a run, in a structure that was defined by the user in the generation phase. In most cases it can be read more easily than the standard output from the instrument. If the results carry error flags, which are attached by the instrument, the evaluator can immediately assess the plausibility of the data. The flags are standardized by the driver program, providing an instrument-independent error indication.

*Data editing*

Experimental results that are obtained either manually or from instruments that cannot be connected with the PC can be input into CAEv for further processing. In the same way, any data item already stored can be altered or eliminated. For editing, the user asks for the data structure of a particular task and requests the data items either arranged by samples or by test codes. All changes to results obtained by online data capture are written to a log file, which allows a trace of the data modifications at the end of a study.

*Processing of results*

Modern instruments can produce numbers of results greater than previously found in evaluation studies in a laboratory, creating logistical problems not only for the

```

Study:                   MC - Study BM/HITACHI 747 (example)

Laboratory:              LAB 10                               Temperature: 37°C

Summary from 23/05/91

Method Comparison

  Hitachi 747

    Series no.  1    ............................  done

    Series no.  2    ............................  done

    Series no.  3    ............................  done

    Series no.  4    ............................  done

    Series no.  5    ............................  done

    Series no.  6    ............................  open

  Coulometry

    Series no.  1    ............................  done

    Series no.  2    ............................  done

    Series no.  3    ............................  done

    Series no.  4    ............................  done

    Series no.  5    ............................  done

    Series no.  6    ............................  open

  AAS

    Series no.  1    ............................  done

    Series no.  2    ............................  done

    Series no.  3    ............................  done

    Series no.  4    ............................  done

    Series no.  5    ............................  done

    Series no.  6    ............................  open

Linearity

  Hitachi 747

    Test code  ASAT  ..........................  done

    Test code  CHOL  ..........................  done

    Test code  FE    ..........................  done

    Test code  NA    ..........................  done

    Test code  K     ..........................  done

    Test code  CL    ..........................  done

Drift

  Hitachi 747
    Series no.  1    ..........................  cancelled

Sample C-O

  Hitachi 747
    Test code  ASAT  ..........................  done
    Test code  FE    ..........................  done
    Test code  K     ..........................  open
    Test code  CL    ..........................  done

Interference

  Hitachi 747
    Selection icteric  ..............................  open
```

*Figure 3. Part of the computer print out from the summary of a (hypothetical) multicentre study.*

handling but also for the statistical processing of the data. CAEv provides tools for the statistical evaluation that access the internal data structures. Therefore, the user can call a statistical evaluation program straight after the completion of the measuring process without any additional data manipulation. This immediate assessment of the laboratory performance allows repetition of measurements and corrective actions, if necessary, before a study is finished and the results are finalized.

It is the aim that the procedures used for the statistical processing of the data cover all relevant evaluation modules or enable data export to appropriate statistical software. In addition, facilities for graphical presentation of the data are desirable. The present CAEv version largely meets these requirements, apart from the graphic output, for which the concept is not yet finalized. Most likely a link to a standard spread-sheet program will be established.

*User support and plausibility*

Any complex application program like CAEv needs extensive support capabilities to ensure a correct usage and reliable output. Potential error sources can arise in the modelling of a study, in the recording of the experimental data (online/offline) and the result processing. A program, therefore, should provide features of an expert system, communication standards with extensive error protocols and facilities for an automatic data validation.

CAEv will provide this kind of support in many various ways. In the generator part the user may call functions that present typical study protocols reflecting the proposals published by standardization institutions. For the underlying biometrical models, the program can offer sample sizes based on the known variability of the data and predefined decision criteria.

In the laboratory part, the quality of communication with the instrument may be monitored and all error conditions that occur during a run can be documented. Automatic sample identification, for example barcode reading, should be supported and the channel or method assignment should be checked automatically.

The validation of results depends to a large extent on the individual experiments. The program could, for example, test the results for deviation from predefined limits or ranges, monitor changes in calibration levels or indicate implausible CVs. To maintain flexibility, CAEv will allow the user to define individual test criteria for a given experiment at the time of study generation.

In the past, not much attention was paid to utilize facilities for a standardized documentation of a study. CAEv offers an easy way to integrate the study definitions and the study results into one document, making use of individual and preformulated blocks of text.

### Program development

The development required a number of important decisions in the planning phase. Two of these were the definition of minimal hardware standards and the selection of a programming language with a suitable developer environment. The hardware decision for a personal computer running under MS-DOS was because this is the computer one will generally find in laboratories. In addition, the use of portable PCs allows an exclusive assignment of hardware to a particular study.

The program was designed to run under the following minimal hardware requirements:

> PC, IBM-AT compatible with minimum of 640 KB RAM, hard disk (20 MB), 1 floppy drive, 1 parallel and 2 serial ports, monochrome or colour display.

For the program development the following were selected:

> Turbo Pascal (Versions 5·5 and 6·0 respectively, Borland International, Inc.), Turbo Professional (Turbo Power Software), B-Tree Isam (Enz, EDV-Beratung GmbH).

During the development of CAEv, special attention was given to the program structure, the user interface, the data management and the instrument/host communication.

*Program structure*

To obtain a program code that is easy to maintain and document, it was decided to work with logically separated modules or topics like generator, laboratory, data management, statistics, communication or user support. This offers the advantage that a given topic can be extended or replaced without affecting the performance or the remaining parts of the program. However, to the user, CAEv appears as a uniform system allowing access to every program module in a transport and consistent way.

*User interface*

Since CAEv is intended to be used by people with different data processing backgrounds, it is important to have an interface between user and program that is easy to comprehend and to remember. For such an objective, the utilization of window and menu techniques is an obvious choice (figure 4). In view of the minimal hardware requirements the user interface was implemented on the basis of character-orientated windows and to stay close to the standards proposed for the System Application Architecture (SAA) by IBM [10]. The resulting program offers a familiar environment to the user, with a good speed performance. In addition, windows and menu driven interfaces are considered an important aid for an occasional user.

The implementation of help functions for context sensitive support is a further contribution to a modern user interface. CAEv provides these functions on the level of

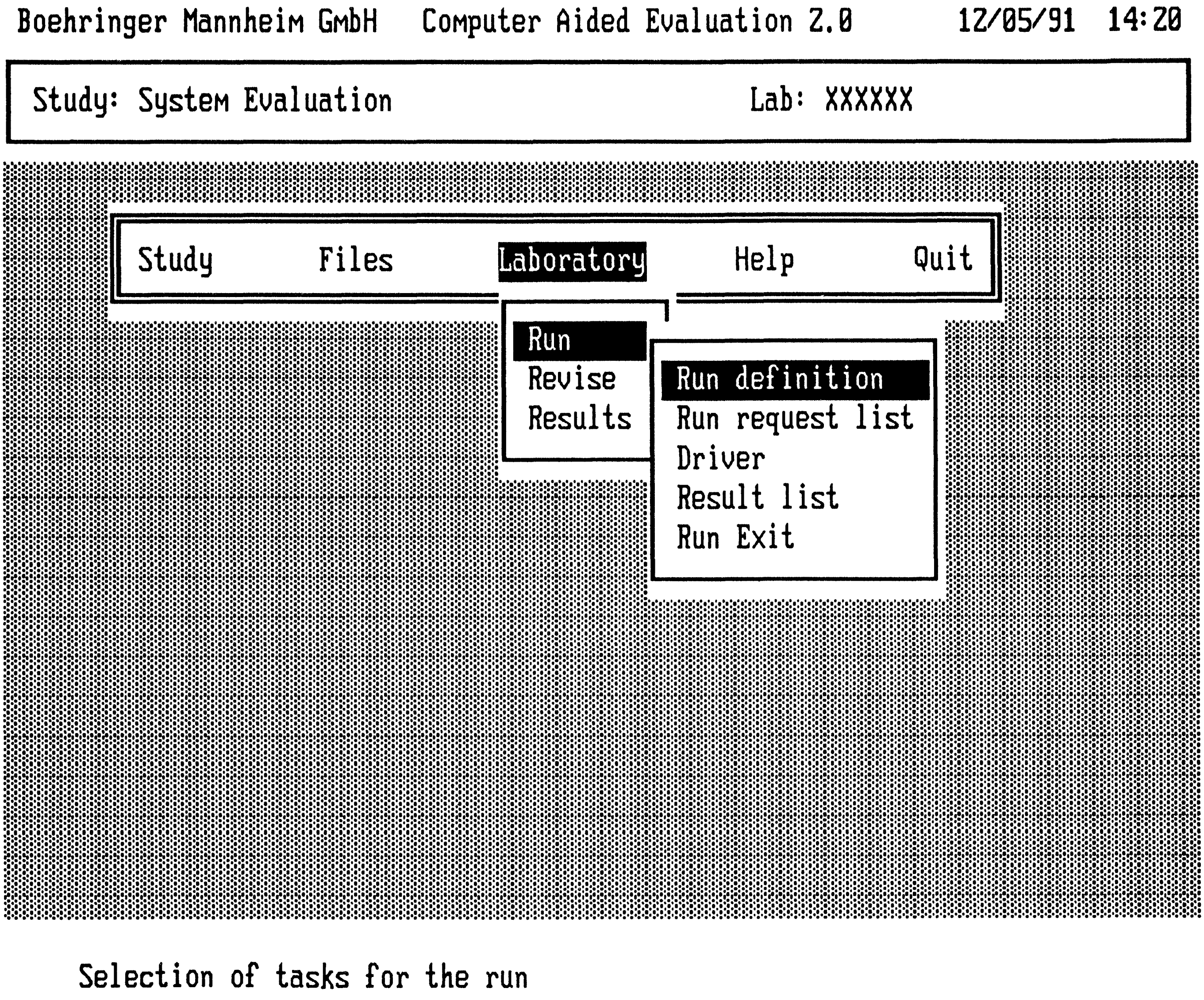

*Figure 4. User interface showing menu bar and sample menus.*

program usage for every step the user can execute, and on the study level for data acquisition and result processing. In addition, it supports the creation of study help texts in the generation phase.

*Data management*

The performance of a program depends to a large extent on an efficient data management. CAEv maintains separate file structures of the generator and laboratory part. Every study and the library files own their individual directory; therefore a file name had to be unique only within such a directory. The tasks/experiments defined for a laboratory in the generator are kept in a condensed form and are transferred directly or by diskette to the PC, which is connected to the analyser. Only when the user defines a run on the instrument, or starts to input data manually, will the data structures be expanded to their full length. Data obtained in a laboratory in the course of a study can be saved at any time in a compressed format for archiving, allowing a reinstallation of the study in the case of file/data corruption or transfer to a central computer for further processing.

*Instrument/host communication*

The driver concept implemented in CAEv for the information exchange with the instruments contributes essentially to the flexibility one should expect from a general application program. It offers a standard data interface on one side and an instrument-orientated communication process on the other.

The communication touches some very important aspects of standardization in the clinical laboratory or hospital environment: hardware interfaces on instrument and computers, use of standard network technologies or of special medical buses [11] and finally the communication protocols between any two instruments. The rapid development in microprocessor technology has produced a heavy demand for the exchange of clinical data for medical and scientific purposes. Even though there are a great number of activities [12] concerned with standardization, common standards for communication in the laboratory are not yet available. Hardware and software standards developed and proposed by the International Standardization Organization (ISO) could be the subject of a future paper.

*Requirements*

In order to establish the communication between an instrument and a host computer, appropriate hardware and software protocols are needed. They are described in the seven-layer reference model, the so-called OSI (Open System Interconnection) model proposed and officially standardized by ISO [13]. In modern analysis systems, several of these layers are implemented, but unfortunately not in a unified way.

In order to avoid a completely new program design for every instrument, the driver was standardized by utilizing all seven layers of the OSI model for the communication part and an instrument-independent ASCII file for the data interface to the CAEv program.

*Data interface*

To develop a general data interface for all possible instruments is a rather complex task. The interface was restricted to instruments which are used in clinical chemistry, and the following data types were considered:

(1) Calibration.

(2) Controls.

(3) Absorbances.

(4) Concentrations (routine measurements).

Basically, each data type is associated with an individual ASCII file. Since controls are not assayed separately on almost all instruments, they are part of the routine sample processing and therefore not kept in a special file. The interface expects digitized output from the instrument and does not provide an analogue to digital conversion.

*Driver functions*

The instrument drivers are designed to use MS-DOS as operating system. Since MS-DOS does not support multitasking, an interrupt driven communication via the serial port is necessary. The drivers, including the interrupt handling, are written in Turbo Pascal. The development of new instrument drivers depends on the demand arising from indivdual evaluation studies. The presently available set of drivers (BM/Hitachi 704, BM/Hitachi 717, BM/Hitachi 747, Cobas Fara/Mira) reflects the requirements of the current users.

From the various functions implemented in the drivers, the database management, the monitoring and the bidirectional communication are briefly discussed.

*Database management:* The driver program maintains information on communication parameters and on channel or method assignment in its own database. In order to allow for an independent definition of test codes in the generator, the driver provides a mapping table, in which the test codes used in CAEv have to be assigned to instrument-specific requirements, for instance channel or method number. Before the driver transmits any request to the instrument, the input data are compared with the database to ensure consistency and completeness.

The communication environment regarding baud rate, port number, barcode, etc. can also be defined and stored for later use.

*Monitoring:* The monitoring of the data transfer between instrument and host is a useful tool for testing the online connection. The driver offers two levels of monitoring, one which presents the sample request and the resulting data from the measuring process and one which documents the complete sequence of the data exchange. This communication trace is displayed on the screen and stored in a circular file, which can be accessed at any time.

*Bidirectional communication:* Modern multichannel analyser systems can obtain the sample request from the host computer and allow different modes for the transfer, for example single or batch request. If the experiment does not require a predefined sample sequence, the use of barcodes enables a direct (positive) sample identification and random sample placement. The current microprocessor technology permits short response times when needed for this kind of operation.

Of importance is the master/slave mode, which controls the communication between instrument and host. All possible combinations can be found in practice. Usually, the analyser operates as master expecting the host to listen constantly. The drivers developed so far are running in this kind of mode.

*Program status*

Version 1 of the CAEv program is available in a very stable state. The program development of version 2 has started. It is the aim of the developers that this version will cover all of the features described in this paper. The main differences will be:

(1) More user comfort in the generator module.

(2) Extension of tasks (recovery, absorbance/time diagrams, etc.).

(3) Easier file handling.

(4) Enhanced user guidance.

The final software package can be made available to evaluators. For further information you should contact the first author.

### Field test

CAEv, version 1, was applied for the first time in the multicentre study of BM/Hitachi 747 [9]. Experience with EVALAB, the laboratory module of the program, is reported from the user's point of view.

*BM/Hitachi 747 evaluation*

BM/Hitachi 747 is a selective access analyser with a capacity of 35 test channels including three ISE methods. The maximum throughput is 3300 tests per hour. The system consists of an analytical unit and a data processing unit, which are linked with an IEEE-488 interface. The data processing unit is a personal computer with Intel 80386 CPU and provides an RS-232-C (serial) interface for communication with the host system.

The evaluation protocol followed mainly the ECCLS guidelines for the evaluation of analysers in Clinical Chemistry [2]. The protocol was agreed upon by the evaluators; it contained a common program with individual components regarding analytes and instruments. Using the generator part of CAEv the complete study with all experiments was defined and prepared for use in the laboratories by the co-ordinator of the multicentre evaluation. The evaluation took place at four different European laboratories. The program covered three phases:

(1) Familiarization.
(2) Initial trial.
(3) Main trial.

During the three-month evaluation period, more than 60 000 data items were recorded.

*Application of CAEv*

The EVALAB part of the CAEv system was distributed before the familiarization period. The software package was installed on a laptop personal computer, which was available to the laboratory for the time of the study. It contained the program and the data files needed at the site of evaluation.

*General features*

After starting CAEv and selecting the study name the main menu is presented to the operator. This menu allows to access four submenus for data processing—*'Evaluation', 'Data-Edit', 'Results', 'Files'*,—and four other submenus for miscellaneous topics like *'Summary'* or *'Help'*.

From the very beginning the program could be handled very easily. If the operator is familiar with PC-based user software, then there is no need to study the program manual extensively. The user-friendliness of the program is supported by

(1) A self-explanatory program structure.
(2) Window techniques and pull-down menus with access to individual topics, and therefore no need to learn a specific command language.
(3) Context-sensitive help screens.

*Data acquisition*

The user can obtain results from experiments either online by a communication link to the instrument or by manual input.

The most convenient way is online data capture, which was used for all experiments running on the BM/Hitachi 747. The program allows to compile more than one experiment into a single run on the instrument. It produces a run request list to support the operator preparing the samples and fitting the sample trays. After accessing the *'Driver'* entry in the *'Run'* submenu the online communication with the analyser is established. The requests are passed to the instrument and the results received either in realtime or in batch mode.

During the study only the realtime mode was used for communication. This mode worked very reliably.

Three comparison instruments were used for the study. The data from these instruments were input manually into the predefined data files.

Offline data recording is selected in the menu *'Data-Edit'* in a sample-orientated or in a testcode-orientated mode, both allowing easy input of new data or correction of existing data. Because of the documentation features and the capabilities for data exchange, CAEv is also useful for evaluation studies, which allow manual input only.

*Plausibility, validation and documentation*

One of the most striking advantages of CAEv, from the user's point of view, is the capability of providing a quick and easy data access during the experiment or immediately after the experiment has been finished. Inspecting the data enables the user to assess the actual results of the experiment and—if necessary—to perform early trouble-shooting. This allows a more effective and economic evaluation.

EVALAB supports topics with different features:

(1) Sample-orientated lists with alphanumeric results.
(2) Specific presentations for different experiments (for example imprecision, linearity, drift, carry over, interference), including results of statistical calculation (figure 5).
(3) Graphical presentation of method comparision.

The features available were adequate for the authors' needs. Nevertheless, an extension of the graphics available would be desirable, because a graphical display of data is more useful for the assessment than a simple print-out.

### General assessment

A thorough evaluation of a large laboratory analyser has become a formidable task. The evaluation process is time-consuming, ties up laboratory personnel and requires, at least in the case of a multicentre trial, an efficient central organization and administration.

Before the introduction of CAEv, a multicentre evaluation could require as long as a year before the results were available for final publication. This is difficult for the company whose instrument is being tested, because,

MC - Study BM/HITACHI 747 Interference by lipemia 29/05/90

Lab: LAB 2 Intr: Hitachi 747 Temp: 25C

page 1

| | PAMYL | | ASAT | | CK | | GGT | |
|---|---|---|---|---|---|---|---|---|
| Base value | 69.0 | | 36.2 | | 399.0 | | 73.8 | |
| TG | | %Rec. | | %Rec. | | %Rec. | | %Rec. |
| 289.0 | 69.0 | 100.0 | 36.2 | 100.0 | 400.0 | 100.3 | 73.8 | 100.0 |
| 433.0 | 70.0 | 101.4 | 36.5 | 100.8 | 400.0 | 100.3 | 74.6 | 101.1 |
| 551.0 | 70.0 | 101.4 | 34.7 | 95.9 | 402.0 | 100.8 | 74.6 | 101.1 |
| 677.0 | 71.0 | 102.9 | 36.2 | 100.0 | 400.0 | 100.3 | 74.6 | 101.1 |
| 814.0 | 69.0 | 100.0 | 35.3 | 97.5 | 400.0 | 100.3 | 74.6 | 101.1 |
| 953.0 | 71.0 | 102.9 | 36.2 | 100.0 | 400.0 | 100.3 | 74.6 | 101.1 |
| 1080.0 | 71.0 | 102.9 | 35.3 | 97.5 | 400.0 | 100.3 | 74.6 | 101.1 |
| 1203.0 | 72.0 | 104.3 | 31.1 | 85.9* | 400.0 | 100.3 | 73.8 | 100.0 |
| 1333.0 | 70.0 | 101.4 | -0.9 | -2.5* | 401.0 | 100.5 | 73.8 | 100.0 |
| 1464.0 | 71.0 | 102.9 | -1.5 | -4.1* | 400.0 | 100.3 | 73.8 | 100.0 |

* deviation from 100% recovery > 10%

*Figure 5. Part of the computer print out from the statistical evaluation of an interference experiment.*

ideally, the instrument should not be marketed before the final approval of the results and their publication. The main purpose of a multicentre-multinational evaluation, after all, is to save many days and weeks of evaluations in individual laboratories, which intend to buy the instrument. This, in turn, is only possible if the evaluation is both timely and highly reliable. Speed and reliability are greatly enhanced by CAEv, and this kind of computer assistance can certainly play a major role in future evaluations.

Although big laboratories of the kind that would make use of large analysers always have access to a laboratory information system (LIS), it is impractical or even impossible to connect the analyser under evaluation to the laboratory computer during the evaluation period. Even if the instrument could be linked to the LIS, communication protocols and communication software need to be available for communication with the central organization. Standard communication protocols, linking several laboratory computers to the statistics centre in a multicentre-multinational evaluation, do not exist and are waiting to be developed.

When examined without the assistance of a computer system, an evaluation differs greatly from routine conditions because routine work practically always uses the computer power for sample identification, test requisition and transfer of results and error flags. Conventional evaluations therefore become more time-consuming and error-prone than routine operations. With the use of CAEv sample identification, definition of test panels and data transfer can be managed much in the same way as in routine operation.

Although the instrument under evaluation was not connected to the LIS, the operation of CAEv in conjunction with the analyser gives a good indication of the performance of the instrument's interface and data-handling software. Prior to the introduction of CAEv, there was no easy way to check interface and communication software of the analyser.

Immediate statistical evaluation of laboratory results at the end of a working day is an important feature that is invaluable for the early detection of errors and malfunctions. This capacity is especially important in method comparision, where deviant results are often treated as outliers without performing a rerun for further testing of the sample.

CAEv can be applied to evaluation and research. It allows the user to define individual standards with respect to study protocols or study tests. The library concept enables the utilization of standard protocols, which can easily be modified for an individual task. The design of the data interface with the communication drivers—which are instrument-specific—means that CAEv is practically independent of the individual properties of an instrument. The safe handling of large data volumes and the immediate access to the experimental results are features of CAEv which greatly enhance the efficiency of the laboratory work.

### References

1. Protocol for the validation of methods, document B, stage 3, Commission for validation of methods of SFBC. *Ann. Biol. Clin.*, **44** (1986), 686.
2. ECCLS, *Guidelines for the Evaluation of Analysers in Clinical Chemistry*, ECCLS document 3, No. 2 (Beuth, Berlin, Köln, 1986).
3. O'Leary, T. D. and Geary, T. D., *Clinical Biochemistry Reviews*, **11** (1990), 68.
4. Lifshitz, M. S. and de Cresce, R. P., *Laboratory Medicine*, **21** (1990), 367.
5. Haeckel, R., *Journal of Automatic Chemistry*, **2** (1980), 22.
6. Knedel, M., Haeckel, R., Seidel, D., Thiery, J., Vonderschmitt, D. J., Haenseler, E., Hubbuch, A., Stockmann, W. and Völkert, E., *Journal of Clinical Chemistry and Clinical Biochemistry*, **24** (1986), 409.
7. Bayer, P. M., Knedel, M., Montalbetti, N., Brenna, S., Prencipe, L., Vassault, A., Bailly, M., Phung, H. T., Bablok, W., Poppe, W. and Stockmann, W., *Journal of Clinical Chemistry and Clinical Biochemistry*, **25** (1987), 919.
8. Baadenhuijsen, H., Bayer, P. M., Keller, H., Knedel, M., Montalbetti, N., Brenna, S., Prencipe, L., Vassault, A., Bailly, M., Phung, H. T., Bablok, W., Poppe, W. and Stockmann, W., *Journal of Clinical Chemistry and Biochemistry*, **28** (1990), 261.
9. Bonini, P., Ceriotti, F., Keller, F., Brauer, P., Pascual, C., Garcia Beltran, L., Vonderschmitt, D. J., Pei, P., Bablok, W., Opitz, I. and Stockmann, W., in preparation.
10. Berry, R. E., *IBM Systems Journal*, **27** (1988), 281.
11. Franklin, D. F. and Ostler, D. V., *IEEE Computer Society Press* (Washington DC, USA, 1989), XIX, 641.
12. Elevitch, F. and Boroviczeny, K.-G., *Archives of Pathological Laboratory Medicine*, **109** (1985), 496.
13. International Organization for Standardization, ISO 7498 (1984).

